# Plant-derived extracellular vesicles as a natural drug delivery platform for glioblastoma therapy: A dual role in preserving endothelial integrity while modulating the tumor microenvironment

**DOI:** 10.1016/j.ijpx.2025.100349

**Published:** 2025-06-24

**Authors:** Lishan Cui, Giordano Perini, Antonio Minopoli, Valentina Palmieri, Marco De Spirito, Massimiliano Papi

**Affiliations:** a Dipartimento di Neuroscienze, Università Cattolica del Sacro Cuore, Largo Francesco Vito 1, 00168 Rome, Italy; b Fondazione Policlinico Universitario A. Gemelli IRCSS, 00168 Rome, Italy; c Istituto dei Sistemi Complessi, CNR, Via dei Taurini 19, 00185 Rome, Italy

**Keywords:** Glioblastoma, 3D bioprinting, Blood-tumor barrier, Plant-derived extracellular vesicles, Drug delivery, VEGF-A

## Abstract

Glioblastoma (GBM) is the most aggressive primary brain tumor, with limited treatment options due to the restrictive blood-brain barrier (BBB) and the heterogeneity of the blood-tumor barrier (BTB). Temozolomide (TMZ), the standard chemotherapy, suffers from poor BBB permeability, rapid degradation, and systemic toxicity. Plant-derived extracellular vesicles (PDEVs) have emerged as promising natural nanocarriers, offering biocompatibility, stability, and the ability to cross biological barriers. This study investigates the use of extracellular vesicles from *Citrus limon* L. (LDEs) to encapsulate and deliver TMZ (EVs@TMZ) for GBM treatment.

LDEs were isolated, characterized, and loaded with TMZ via ultrasonication. Encapsulation efficiency, stability, and physicochemical properties were assessed using UV–Vis and FTIR spectroscopy. A 3D BTB model was developed using bioprinted U87 glioblastoma cells in Matrigel, co-cultured with hCMEC/D3 endothelial cells to replicate the tumor microenvironment. Barrier integrity was evaluated through TEER and FITC-dextran assays. Uptake, cytotoxicity, and tumor invasion were assessed in this model, along with oxidative stress and VEGF-A secretion.

LDEs effectively encapsulated TMZ, enhancing drug stability under physiological conditions. EVs@TMZ crossed the endothelial barrier while preserving barrier integrity and reducing TMZ-induced ROS production. In the 3D glioblastoma model, EVs@TMZ showed strong cytotoxic effects on tumor cells while minimizing endothelial toxicity and oxidative stress. Moreover, VEGF-A secretion was suppressed, disrupting pro-tumorigenic pathways.

These findings highlight Citrus-derived EVs as biocompatible, efficient carriers for TMZ delivery, offering a promising approach to overcome current challenges in GBM therapy and supporting further development of PDEVs for brain tumor treatment.

## Graphical abstract

**Figure:**
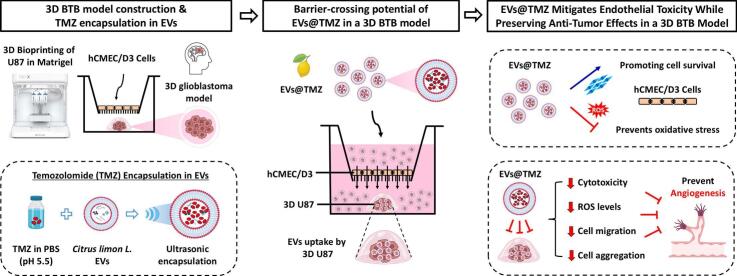

## 1 Introduction

Glioblastoma (GBM) remains one of the most huge challenge in oncology, marked by its aggressive nature, extensive infiltration into surrounding brain tissue, and resistance to conventional therapies (Auffinger et al., 2014). Current treatment strategies, consisting of maximal surgical resection followed by radiotherapy and chemotherapy, have led to gradual improvements in survival outcomes but remain insufficient in achieving long-term disease control. Temozolomide (TMZ), an alkylating agent that induces DNA damage and apoptosis in tumor cells, is the first-line chemotherapeutic agent for GBM treatment. (Wu et al., 2023). Despite its therapeutic advantages, TMZ is associated with considerable limitations, including clinically significant toxicity in approximately 15–20 % of patients, which may necessitate dose reductions or treatment discontinuation, thereby compromising therapeutic efficacy (Chamberlain, 2010).

Beyond intrinsic resistance, the effective delivery of chemotherapeutic agents is further hindered by the unique physiological barriers in the brain. While TMZ is one of the few agents capable of crossing the BBB, its distribution within the tumor is not uniform due to the presence of the blood-tumor barrier (BTB), a pathological structure that forms within and around brain tumors. Unlike the BBB, the BTB exhibits heterogeneous permeability due to disrupted tight junctions and an irregular vascular architecture, resulting in inconsistent drug penetration across different tumor regions. While some regions of the BTB are more permeable than the intact BBB, others remain restrictive, limiting uniform drug distribution within the tumor mass (Cai et al., 2023). Furthermore, the BTB is characterized by the aberrant expression of efflux transporters, such as P-glycoprotein and multidrug resistance-associated proteins, which actively expel chemotherapeutic agents and further reduce their intracellular accumulation (Miller et al., 2008). These factors collectively result in suboptimal drug delivery, necessitating innovative strategies to improve GBM treatment outcomes.

Extracellular vesicles (EVs) have emerged as a promising platform for drug delivery, offering a dynamic and effective approach to overcome biological barriers and enhancing therapeutic efficacy (Elsharkasy et al., 2020). These nanosized vesicles, composed by a lipid bilayer, are naturally secreted by various cell types and serve as key mediators of intercellular communication (Berumen Sánchez et al., 2021). By transporting a diverse array of bioactive molecules (proteins, lipids, RNA, and metabolites), EVs regulate a wide range of physiological and pathological processes, including immune regulation (Zhou et al., 2020), tissue regeneration (Nagelkerke et al., 2021), and tumor progression (Marar et al., 2021). One of the key advantages of EVs as drug carriers is their capacity to overcome biological barriers, particularly the BBB, which poses a major challenge in the treatment of neurological disorders and brain malignancies (Saeedi et al., 2019). As an alternative to synthetic nanoparticles, EVs have enhanced biocompatibility and low immunogenicity, which may facilitate their systemic administration while minimizing adverse immune responses (Witwer and Wolfram, 2021). Their endogenous nature allows for efficient cellular uptake, potentially leading to improved intracellular drug delivery and therapeutic efficacy.

Plant-derived extracellular vesicles (PDEVs), such as those from *Citrus limon L.*, have garnered significant interest due to their unique biological properties and potential therapeutic applications. With advantages over mammalian EVs, PDEVs, exhibit inherent advantages such as low immunogenicity, excellent biocompatibility, and large-scale production, enabling a sustainable supply of substances from edible and medicinal plants (Xu et al., 2023). With favorable safety profiles and cost-effective production, PDEVs present an ethical alternative to mammalian EVs, avoiding the risks of harmful genetic abnormalities, infection, or transfer of undesirable donor cell traits. These advantages make PDEVs promising candidates for innovative drug delivery systems, cancer treatment, and the modulation of cellular pathways in various diseases. Recent evidence suggests that PDEVs can enhance the bioavailability of chemotherapeutic agents (Alzahrani et al., 2023), modulate immune responses (Kürtösi et al., 2024), and facilitate targeted drug transport (Lian et al., 2022), making them a powerful alternative to synthetic nanocarriers.

Building on our previous findings that *Citrus limon L.*-derived EVs promote tissue regeneration and influence tumor progression in triple-negative breast cancer (TNBC), this study explores their potential as nanocarriers for TMZ. We investigated their encapsulation efficiency, physicochemical features, BBB permeability, and therapeutic efficacy in a 3D BTB model. By exploiting the intrinsic properties of PDEVs, this approach aims to enhance TMZ stability, facilitate its transport across the BBB, and improve its targeted delivery to GBM cells while minimizing off-target toxicity. Beyond serving as drug carriers, *Citrus limon L.*-derived EVs exhibit bioactive properties, including anti-inflammatory and antioxidant effects, which may provide synergistic therapeutic benefits in tumor suppression. Overall, their unique features position them as a promising strategy to address the limitations of traditional TMZ delivery, potentially advancing GBM treatment and improving patient outcomes.

## 2 Materials and methods

### 2.1 Isolation and characterization of EVs derived from *Citrus Limon L.*

The isolation and characterization of EVs derived from *Citrus limon* L. juice was performed following previously established protocols (Cui et al., 2024b). Briefly, the juice was centrifuged at 3000 ×*g* for 30 min to remove dead cells, cell debris, and large particles. The resulting supernatant was then centrifuged at 10,000 ×*g* for 60 min to eliminate smaller debris. After filtration through a 0.22 μm pore filter, the supernatant was subjected to ultracentrifugation at 100,000 ×*g* for 90 min using a Type 50.2 Ti fixed-angle rotor (Beckman Coulter Inc., Brea, CA, USA). To enhance the purity of small EVs and separate them from contaminants of similar size and density, the pellet was resuspended in 5 mL of cold PBS (1×) and layered onto a 30 % sucrose/D_2_O cushion. This ultracentrifugation step at 100,000 ×*g* and 4 °C for 120 min allowed the EVs to settle into the cushion due to their density. The nanovesicle-containing fraction was then resuspended in PBS (1×) and subjected to two additional ultracentrifugation steps at 100,000 ×*g* and 4 °C for 90 min each to remove residual sucrose. Finally, the pellet was resuspended in PBS (1×) for subsequent analyses. The size and morphology of the EVs were confirmed by transmission electron microscopy (TEM). Nanoparticle tracking analysis (NTA) using the NanoSight NS300 (Malvern Technologies, Malvern, UK) was employed to determine the concentration and size distribution of the EVs. Protein concentrations were quantified using the bicinchoninic acid (BCA) assay kit (Thermo Fisher Scientific, Waltham, MA) according to the manufacturer's guidelines.

### 2.2 Cell culture

The human brain microvascular endothelial cell line hCMEC/D3 (Catalog #305024) was purchased from Cytion Biosciences. Cells were cultured in EGM™-2 MV Microvascular Endothelial Cell Growth Medium-2 BulletKit™ (Lonza, Basel, Switzerland), prepared according to the manufacturer's instructions. Briefly, the basal medium was supplemented with growth factors and supplements provided in the kit, including vascular endothelial growth factor (VEGF), R3-insulin-like growth factor (IGF-1), human epidermal growth factor (hEGF-B), hydrocortisone, ascorbic acid, and gentamicin/amphotericin B. Human glioblastoma cell line U87 was purchased from American Type Culture Collection (ATCC, Rockville, MD, USA). Cells were maintained in Dulbecco's Modified Essential Medium (DMEM, Sigma-Aldrich, St. Louis, MO, USA) supplemented with 10 % fetal bovine serum (FBS, Gibco, Life Technologies) and 2 % penicillin–streptomycin (Sigma-Aldrich, St. Louis, MO, USA). Cells were maintained at 37 °C with 5 % CO_2_ under a humidified atmosphere.

### 2.3 3D bioprinting-based fabrication of a BTB model

The CELLINK BIO X 3D bioprinter was used to bioprint 5000 cells per drop into Corning Matrigel Growth Factor Reduced (GFR) Basement Membrane Matrix (Cat. #354230). Prior to use, Matrigel was thawed overnight at 4 °C. The cells were trypsinized, resuspended in cold culture medium, and then combined with Matrigel to achieve a final gel concentration of 8 mg/mL. The cell-Matrigel suspension was loaded into pre-cooled printing cartridges, which were attached to the cooled printhead of the bioprinter. The temperature of the print bed was set to 37 °C, and the printhead was maintained at 4 °C. The extrusion rate was set at 5 μL/s, with each drop having a volume of 5 μL. The *Z*-lift between the wells was set to 2.0 mm. After printing, the plate was inverted and incubated at 37 °C for 30 min to allow the Matrigel to polymerize. Subsequently, culture medium was added to each well. hCMEC/D3 cells were co-cultured at a cell density of 100,000 cells/well in a volume of 200 μL on top of 24-well Transwell inserts (0.4 μm pore size).

### 2.4 *Trans*-endothelial electrical resistance (TEER) measurement

TEER values were measured by using Millipore Millicell ERS-2 device (Part number MERS00002). Measurement was performed avoid air bubbles, as these can interfere with resistance measurements. The Transwell plate was placed in an incubator to equilibrate at 37 °C for 30 min before measurement to avoid affecting the resistance readings. Blank resistance was measured with Transwell inserts containing no cells and filled with culture medium only. TEER probes were inserted into the apical and basolateral compartments of the wells containing endothelial cells. The TEER value was calculated by subtracting the blank resistance from the total resistance and then multiplying the net resistance by the surface area of the Transwell insert (0.33 cm^2^ for a 24-well insert). TEER is expressed in ohms·cm^2^, a standardized unit that accounts for variations in the surface area of the inserts.

### 2.5 Fluorescein isothiocyanate (FITC)–dextran permeability assay

Cells were pre-incubated in serum-free medium for 30 min to eliminate potential interference from serum proteins and to stabilize the endothelial barrier. Subsequently, 200 μL of a 1 mg/mL solution of Fluorescein Isothiocyanate (FITC)–dextran (20 kDa) (Sigma-Aldrich, Cat# FD20S) in PBS was added to the apical compartment of the Transwell insert, while the basolateral compartment contained 800 μL of serum-free medium. The plate was incubated at 37 °C, and measurements were taken at 5-min intervals for up to 1 h. Fluorescence intensity was measured at excitation/emission wavelengths of 495/520 nm.

### 2.6 Immunofluorescence analysis of tight junction protein

For immunofluorescence staining, hCMEC/D3 cells were fixed with 4 % paraformaldehyde for 15 min at room temperature (RT), followed by three washes with PBS. Permeabilization was performed using 0.1 % Triton X-100 in PBS for 15 min at RT, and nonspecific binding sites were blocked by incubating cells in 2 % bovine serum albumin (BSA) in PBS for 1 h at room temperature. Cells were then incubated 3 h at RT with polyclonal anti-ZO-1 antibody (Thermo Fisher, #40–2200) diluted 1:100 in blocking buffer (PBS containing 0.1 % BSA). Following three washes, the cells were incubated with Goat anti-Rabbit IgG (Heavy chain) Secondary Antibody, Alexa Fluor 488 (Product # A27034) for 1 h at RT in the dark. Afterward, the cells were washed three times with PBS before being stained with Alexa Fluor 594 Phalloidin (Product # R415) for 1 h at room temperature to label actin filaments. Following the staining, the cells were washed three more times with PBS, and the nucleus were stained with DAPI (4′,6-Diamidino-2-Phenylindole) for 15 min in the dark at room temperature. After three additional PBS washes, the membranes were carefully excised from the Transwell inserts and mounted onto an ibidi μ-Dish 35 mm, high Glass Bottom (ibidi, Germany). Fluorescence images were acquired using a Nikon A1 MP + multiphoton confocal microscope.

### 2.7 Cellular uptake of EVs and EVs@TMZ in 3D U87 cells

EVs were stained with Calcein-AM, while cells were labeled with Rhodamine Phalloidin following previously established protocols (Cui et al., 2024a). 3D models were incubated with labeled-EVs for 24 h at 37 °C. After labeling, samples were fixed with 4 % paraformaldehyde for 30 min at room temperature, followed by three washes with PBS. They were then permeabilized with 0.1 % Triton X-100 (Sigma-Aldrich) for 15 min at room temperature and washed twice with PBS. Finally, nuclei were stained with DAPI (4′,6-Diamidino-2-Phenylindole) for 15 min in the dark at room temperature. After three additional PBS washes, the 3D U87 model was carefully detached from the bottom of the wells and mounted onto an ibidi μ-Dish (35 mm). Cellular uptake of EVs and EVs@TMZ was assessed using confocal microscopy, with 3D fluorescence *Z*-stack images captured on a Nikon A1 MP+ multiphoton confocal microscope equipped with a 60× oil immersion objective.

### 2.8 Stability of TMZ in PBS at different pH Levels

Temozolomide (Cat. No. 466760010) was purchased from Thermo Fisher Scientific. The UV–Vis spectrum of TMZ was measured using a spectrophotometer across a wavelength range of 240–400 nm. TMZ solutions were prepared in DMSO at concentrations ranging from 0 to 1 mg/mL to construct a calibration curve. The absorbance at 328 nm, corresponding to the maximum absorbance peak of TMZ, was used for quantitative analysis. The hydrolytic stability of TMZ was evaluated in PBS at pH levels 7.4, 6.8, 5.5, and 2.5. TMZ solutions were incubated in the respective buffers at 37 °C for 0 to 72 h. At each time point, samples were collected, and their absorbance spectra were recorded. The formation of an isosbestic point was also monitored to assess the degradation pathway. UV–visible spectroscopy readings were performed with a BioTek Cytation 3 Cell Imaging Multimode Reader using BioTek Take3 microplates (Agilent Technologies).

### 2.9 Encapsulation of TMZ into EVs

TMZ was encapsulated into EVs by ultrasonication using a Diagenode Bioruptor 300 equipped with an integrated cooling system to maintain the sample temperature at 4 °C. The protocol consisted of six cycles, each involving 30 s of sonication at low amplitude, followed by a 30-s pause, to ensure uniform energy input while minimizing thermal stress. For the encapsulation process, 10^13^ EVs were mixed with 0.1 mg of TMZ, resulting in a final TMZ concentration of 0.5 mg/mL and EVs concentration of 1 mg/mL in the reaction mixture. After sonication, the mixture was immediately placed on ice for 30 min to cool and recover, and subsequently stored at −20 °C for further analysis.

### 2.10 Quantifying encapsulation efficiency (EE)

The separation of EVs@TMZ from free TMZ was performed using Spin-X UF 500 ultrafiltration concentrators (Sigma-Aldrich, Cat. No. CLS431478) with a molecular weight cutoff (MWCO) of 30 kDa. Prior to use, the filters were sterilized by washing with 75 % ethanol and rinsed once with sterile PBS. To remove residual liquid, the filters were centrifuged at 1000 ×*g* for 2–3 min. The sample mixture containing EVs@TMZ and free TMZ was loaded into the prepared concentrators and centrifuged at 12,000 ×*g* for 10 min. The centrifugal force allowed filtrate (free TMZ), with a molecular weight of 194.15 Da, to pass through the ultrafiltration membrane into the filtrate, while retentate (EVs@TMZ), which exceeded the MWCO, were retained in the upper chamber. The filtrate was collected for quantification of unencapsulated TMZ using UV–Vis spectroscopy. The encapsulation efficiency (EE) was calculated using the following formula:

$$\text{Encapsulation Efficiency}\;\left(\%\right)=\frac{\text{Total}\;\mathrm{TMZ}-\text{Free}\;\mathrm{TMZ}\;\text{in Filtrate}}{\text{Total}\;\mathrm{TMZ}}\times 100$$

### 2.11 Fourier transform infrared spectroscopy (FT-IR) analysis

FT-IR spectroscopy was performed to analyze the chemical composition and confirm the encapsulation of TMZ within EVs. The analysis was conducted using the ALPHA II compact FT-IR spectrometer (Bruker Optics, Ettlingen, Germany) equipped with a Platinum attenuated total reflectance (ATR) module. Lyophilized samples, including free TMZ, blank EVs, and EVs@TMZ, were placed on ATR crystal. The contact between the samples and the crystal was ensured using the built-in pressure applicator. Spectra were recorded over the range of 4000–400 cm^−1^ at a resolution of 4 cm^−1^, with an average of 32 scans per sample to improve the signal-to-noise ratio. Background spectra were recorded prior to each measurement to eliminate environmental interference. Key functional groups and characteristic peaks were analyzed to identify chemical interactions and confirm the successful encapsulation of TMZ within EVs. Spectral data were processed using OPUS software (Bruker).

### 2.12 Drug release study

To evaluate the release of TMZ from EVs@TMZ, the phospholipid membrane of EVs was lysed. Triton X-100, a nonionic surfactant, was chosen to selectively permeabilize or disrupt the lipid bilayer of the EVs, enabling the release of encapsulated TMZ. For the experiment, the EVs@TMZ mixture was incubated with 0.1 % (*v*/v) Triton X-100 at RT for up to 30 min. The reaction was gently mixed to ensure uniform exposure of the EVs to the surfactant. Samples were collected at predetermined time points (e.g., 10, 20, and 30 min) to monitor the release kinetics of TMZ.

### 2.13 Calcein AM − PI live/dead assay

Live/dead cell staining was carried out using Calcein AM and propidium iodide (PI). hCMEC/D3 and U87 cells (1 × 10^4^ per well) were seeded in a 96-well plate with complete medium and incubated overnight at 37 °C in a 5 % CO₂. The cells were then incubated with EVs at concentrations of 10, 20, 40, and 80 μg/mL for 48 h. Afterwards, cells were stained with the green-fluorescent Calcein AM for 20 min, followed by a 5-min incubation with PI. Fluorescence images were captured using a Cytation 3 Cell Imaging Multi-Mode Reader, with excitation at 488 nm and emission detected at 525 nm.

### 2.14 CellTiter-Glo 2D luminescent cell viability assay

The viability of hCMEC/D3 and U87 cells was assessed using the CellTiter-Glo Luminescent Cell Viability Assay (Promega, Madison, WI, USA). A total of 1 × 10^4^ cells were seeded in each well of a 96-well plate with complete medium and incubated overnight at 37 °C in a 5 % CO₂. After 24 h, the cells were treated with EVs at graded concentrations of 10, 20, 40, and 80 μg/mL. Following a 48-h incubation at 37 °C, the cells were carefully washed with PBS. An equal volume of CellTiter-Glo reagent was then added to each well and mixed with the culture medium. The plate was placed on an orbital shaker for 2 min to facilitate cell lysis, followed by a 10-min incubation at RT in the dark to stabilize the luminescent signal. The luminescence was subsequently measured using Cytation 3.

### 2.15 CellTiter-Glo 3D cell viability assay

Cell viability in the 3D models was assessed using the CellTiter-Glo 3D Luminescent Cell Viability Assay (Promega, Madison, WI, USA). CellTiter-Glo 3D Reagent efficiently permeates spheroids, allowing for accurate quantification of 3D cytotoxicity. The 3D bioprinted glioblastoma models were cultured in complete DMEM medium and incubated overnight at 37 °C and 5 % CO₂. The following day, the 3D models were treated with either TMZ or EVs@TMZ for 48 h. After incubation, the cells were thoroughly washed with PBS. Subsequently, an equal volume of CellTiter-Glo 3D reagent was added to each well, followed by gentle agitation for 5 min to ensure complete cell lysis. The plate was subsequently incubated at RT in darkness for 25 min to stabilize the luminescence signal. Luminescence intensity was recorded using Cytation3.

### 2.16 Cellular reactive oxygen species (ROS) detection assay

Reactive oxygen species (ROS) generation was assessed using the fluorinated analog of 2′,7′-dichlorofluorescein (H₂DCFDA; Sigma-Aldrich), a cell-permeable, non-fluorescent probe. Once inside cells, H₂DCFDA is deacetylated by intracellular esterases to form H₂DCF, which is then oxidized by ROS to generate highly fluorescent 2′,7′-dichlorofluorescein (DCF). For ROS detection, cells were incubated with H₂DCFDA at a final concentration of 10 μM and maintained at 37 °C for 30 min to allow uptake and oxidation. Following incubation, the staining solution was removed, and fresh medium was added to eliminate background fluorescence. The fluorescence intensity of DCF was measured using a microplate reader, with excitation at 495 nm and emission at 525 nm. The detected ROS levels were normalized to the number of viable cells to ensure accurate quantification.

### 2.17 Wound healing assay

A total of 1 × 10^5^ hCMEC/D3 cells were seeded into a 24-well plate and incubated overnight at 37 °C in a 5 % CO₂ incubator. The following day, upon reaching approximately 90 % confluence, a sterile P-200 pipette tip was used to create a scratch in the cell monolayer. Cells were then washed twice with prewarmed (37 °C) PBS to remove detached cells and debris while minimizing accidental cell shedding. Subsequently, cells were incubated for up to 24 h in the presence or absence of EVs. Wound healing progress was continuously monitored at 4× magnification using a Cytation 3 Cell Imaging Multifunction Reader, and the area of wound closure was quantified using ImageJ software.

### 2.18 Enzyme-linked immunosorbent assay (ELISA)

The levels of vascular endothelial growth factor A (VEGF-A) were quantified using a Human VEGF-A ELISA Kit (Sigma-Aldrich, RAB0507) according to the manufacturer's instructions. Briefly, all reagents and samples were brought to RT prior to use. Cell culture supernatants were stored at −80 °C to prevent degradation, avoiding repeated freeze-thaw cycles. A standard curve was prepared by serially diluting the recombinant VEGF-A standard in the provided diluent buffer, covering a concentration range of 8.23–6000 pg/mL. For each well, 100 μL of standards or samples were added and incubated at RT. After 2.5 h of incubation, wells were washed three times with wash buffer to remove unbound components. Next, 100 μL of biotinylated detection antibody was added to each well, followed by a 1-h incubation and subsequent washing. Streptavidin-HRP conjugate (100 μL) was then added, incubated for 1 h, and thoroughly washed. The colorimetric reaction was initiated by adding 100 μL of substrate solution to each well and incubating the plate in the dark for 1 h. At the end, the reaction was stopped with 50 μL of stop solution, and optical density (OD) was measured at 450 nm using a microplate reader. The VEGF-A concentration in the samples was calculated by interpolation of the OD values of the standard curve.

### 2.19 Statistical analysis

Quantitative data were obtained from three independent experiments and are presented as the mean ± standard error of the mean (SEM). Statistical significance was evaluated using one-way or two-way analysis of variance (ANOVA), depending on the experimental design. Tukey's post hoc test was applied for multiple comparisons to determine significant differences between groups. All statistical analyses were conducted using GraphPad Prism 9 (GraphPad Software, San Diego, CA, USA). A *p*-value less than 0.05 was considered statistically significant, and the significance levels were as follows: *p < 0.05, **p < 0.01, ***p < 0.001, and ****p < 0.0001.

## 3 Results

### 3.1 Construction of a 3D BTB model to explore the barrier-crossing potential of *Citrus limon L.*-derived EVs

**Fig. 1:**
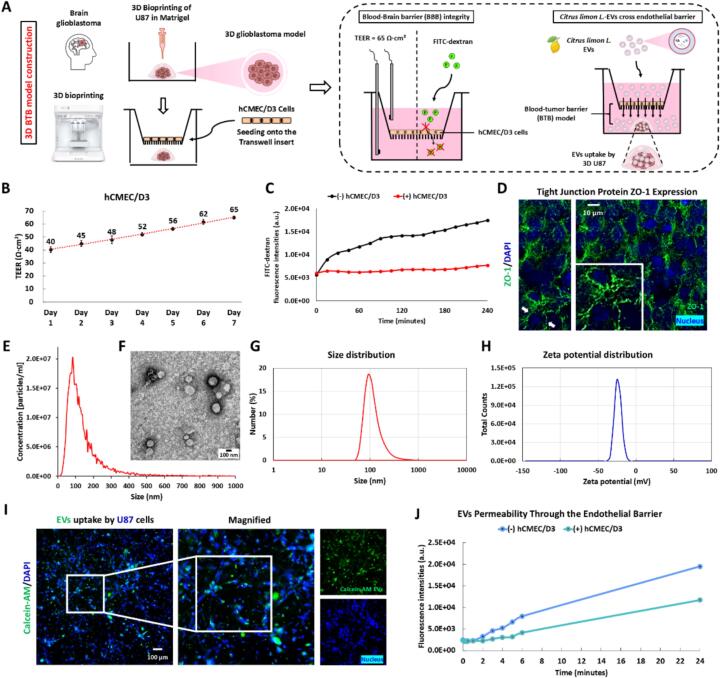
Construction of a 3D BTB model and assessment of the barrier-crossing ability of *Citrus limon L.*-derived EVs. (A) Schematic representation of the 3D BTB model construction and EVs permeability assessment. (B) TEER measurements assessing endothelial barrier formation in hCMEC/D3 cells over seven days. (C) FITC-dextran permeability in the absence (−) or presence (+) of hCMEC/D3 cells. (D) Immunofluorescence staining of the tight junction protein ZO-1 (green) in hCMEC/D3 cells. Nuclei are stained with DAPI (blue). (E) Nanoparticle tracking analysis (NTA) of size distribution and concentration of EVs. (F) Representative transmission electron microscopy (TEM) image of EVs (Scale bar = 100 nm). (G) Size and (H) zeta potential distribution of *Citrus limon L.*-derived EVs. (I) Fluorescence images showing the uptake of Calcein-AM-labeled EVs (green) by U87 glioblastoma cells. Nuclei are stained with DAPI (blue). scale bar = 100 μm. The magnified images depict high-magnification views of the white boxed areas. (J) Fluorescence intensities of endothelial barrier-crossed EVs were measured using a Cytation 3 Cell Imaging Multi-Mode Reader (BioTek, Winooski, VT, USA). Error bars represent the standard deviation (or standard error) of the mean; however, they are shorter than the height of the symbols and therefore not visible in the figure. (For interpretation of the references to colour in this figure legend, the reader is referred to the web version of this article.)

To mimic the BTB microenvironment and construct a 3D GBM tumor model, this study designed an innovative 3D co-culture strategy based on the transwell system. The top transwell chamber represents the “blood” side, with hCMEC/D3 cells cultured on the membrane, and the bottom chamber represents the tumor side, 3D U87 glioblastoma cells in Matrigel to reproduce physiologically relevant 3D tumor microenvironment (Fig. 1A). The integrity of the BBB (top level) was assessed by trans-endothelial electrical resistance (TEER) and FITC-dextran permeability assays. The progressive increase in TEER values over seven days suggests the successful establishment of a mature and functional endothelial barrier, aligning with reported in vitro BBB models (Weksler et al., 2013). The observed TEER values for hCMEC/D3 monolayers (∼65 Ω·cm^2^) suggest that adequate tight junctions were formed (Fig. 1B). Consistently, permeability assays revealed a significant reduction in FITC-dextran diffusion in the presence of endothelial cells, further confirming barrier integrity (Fig. 1C). The immunofluorescence analysis of ZO-1 expression provides additional validation, demonstrating well-organized tight junction, which is critical for maintaining the restrictive nature of the BBB (Fig. 1D). To further support these findings, immunofluorescence staining was performed to visualize the cytoskeletal network in hCMEC/D3 monolayers. As shown in Supplementary Fig. 4, actin filaments (F-actin) stained with Rhodamine-phalloidin (red), highlight the structural organization of the endothelial barrier. Co-localization of ZO-1 (green) with the actin cytoskeleton emphasizes the role of cytoskeletal dynamics in stabilizing endothelial junctions, further enhancing the functional integrity of the BBB. The barrier-crossing ability of the EVs derived from *Citrus limon* L. was evaluated, as well as their uptake by U87 cells. The size distribution of EVs, as determined by nanoparticle tracking analysis and dynamic light scattering, shows a predominant population around 100 nm (Fig. 1E&G). Transmission electron microscopy images confirmed the presence of spherical morphology (Fig. 1F), further supporting their classification as exosome-like structures. Zeta potential measurements revealed a negative surface charge (∼ −25 mV) (Fig. 1H), indicative of colloidal stability and consistent with previous reports on plant-derived extracellular vesicles (Lian et al., 2022). The internalization of Calcein-AM-labeled EVs by U87 glioblastoma cells confirms their ability to be taken up by mammalian cells (Fig. 1I). The efficient cellular uptake supporting their potential as natural nanocarriers for therapeutic applications. The permeability assay reveals that *Citrus limon L.*-derived EVs successfully traverse the endothelial barrier, with fluorescence intensity increasing over time (Fig. 1J). These findings agree with reports on EVs transport across biological barriers, where their small size and lipid composition facilitate transcytosis across endothelial monolayers (Ramos-Zaldívar et al., 2022). The ability of these vesicles to traverse the brain endothelial barrier highlights their potential for targeted drug delivery to glioblastoma cells, reinforcing the emerging role of PDEVs in overcoming physiological barriers in brain tumors.

### 3.2 Cellular uptake of EVs in 3D glioblastoma cells and TMZ drug response

**Fig. 2:**
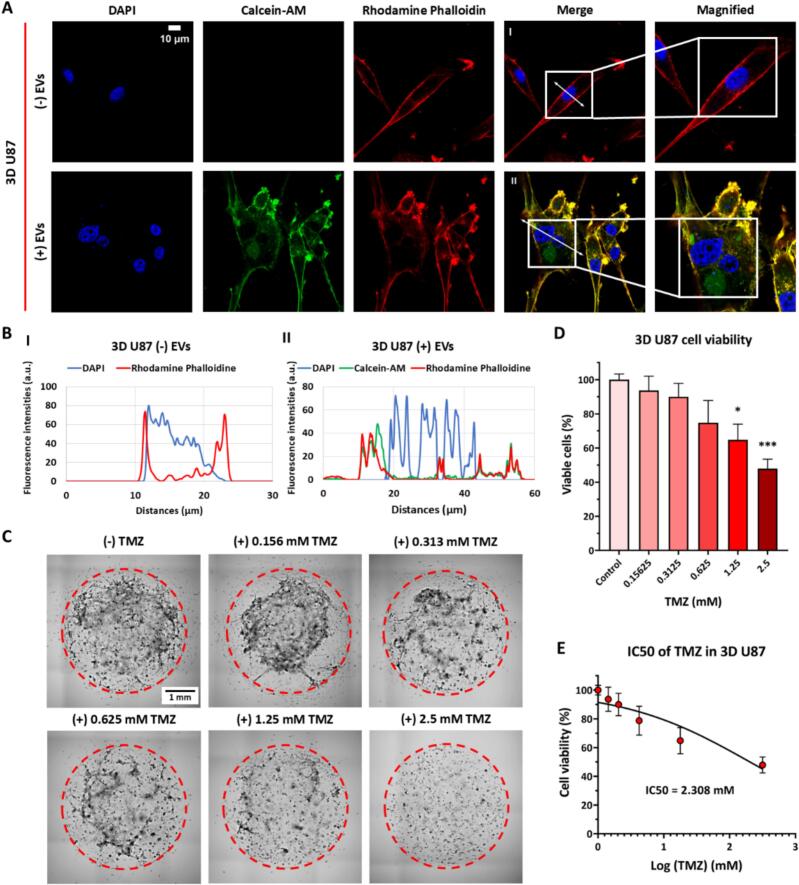
EVs Uptake by 3D U87 glioblastoma cells and the cytotoxic effects of TMZ in the 3D model. (A) Representative images of 3D U87 cells incubated with (+) or without (−) EVs at 37 °C for 24 h. EVs were labeled with calcein-AM (shown in green), actin filaments were labeled with rhodamine phalloidin, and nuclei were counterstained with 4′,6-diamidino-2-phenylindole (DAPI) (scale bar = 10 μm). The magnified images depict high-magnification views of the white boxed areas. (B) Fluorescence intensity profiles of cellular components under (−) EVs and (+) EVs conditions. (C) Representative stitched microscopic images of 3D U87 after 48 h of TMZ treatment ranging from 0 to 2.5 mM (Scale bar = 1 mm). (D) Dose-dependent cytotoxicity of TMZ on 3D U87 model. (E) IC50 of TMZ in 3D U87 model. The IC50 value is fitted from sigmoidal dose-response curve to quantify drug potency. Bars, mean ± SEM. Statistical significance was evaluated using one-way ANOVA followed by Tukey's post-hoc tests (*p < 0.05; **p < 0.01; ***p < 0.001). (For interpretation of the references to colour in this figure legend, the reader is referred to the web version of this article.)

Recent studies have highlighted the advantages of 3D tumor models in mimicking the in vivo tumor microenvironment, particularly in glioblastoma, where the extracellular matrix and cell-cell interactions play a crucial role in drug response and resistance (Gomez-Roman et al., 2017). Given the emerging role of PDEVs as bioactive nanocarriers with potential therapeutic effects, we investigated their efficient uptake in 3D glioblastoma cells. Fluorescence microscopy images show the presence of Calcein-labeled EVs within the cytoplasm, co-localizing with Rhodamine-Phalloidin-stained actin filaments, indicating active cellular interaction, highlighting the potential of EVs as nanocarriers in glioblastoma treatment (Fig. 2A). Quantitative fluorescence intensity profiling further supports this observation, demonstrating the spatial distribution of EVs within the cells (Fig. 2B). To evaluate the cytotoxic effects of TMZ in the 3D U87 model, the cells were exposed to increasing concentrations of the drug. Representative images reveal the dose-dependent cytotoxic effect of TMZ, with progressive inhibition of cancer cell proliferation (Fig. 2C). Quantitative viability analysis further confirms this trend, with a significant cytotoxic effect observed at concentrations above 1.25 mM (Fig. 2D). By determining the half-maximal inhibitory concentration (IC50) of TMZ (2.31 mM) (Fig. 2E), we gained insight into the therapeutic response of 3D GBM cells, emphasizing the relevance of 3D tumor models in drug efficacy testing and optimization of glioblastoma treatment.

### 3.3 pH- and time-dependent stability of TMZ

**Fig. 3:**
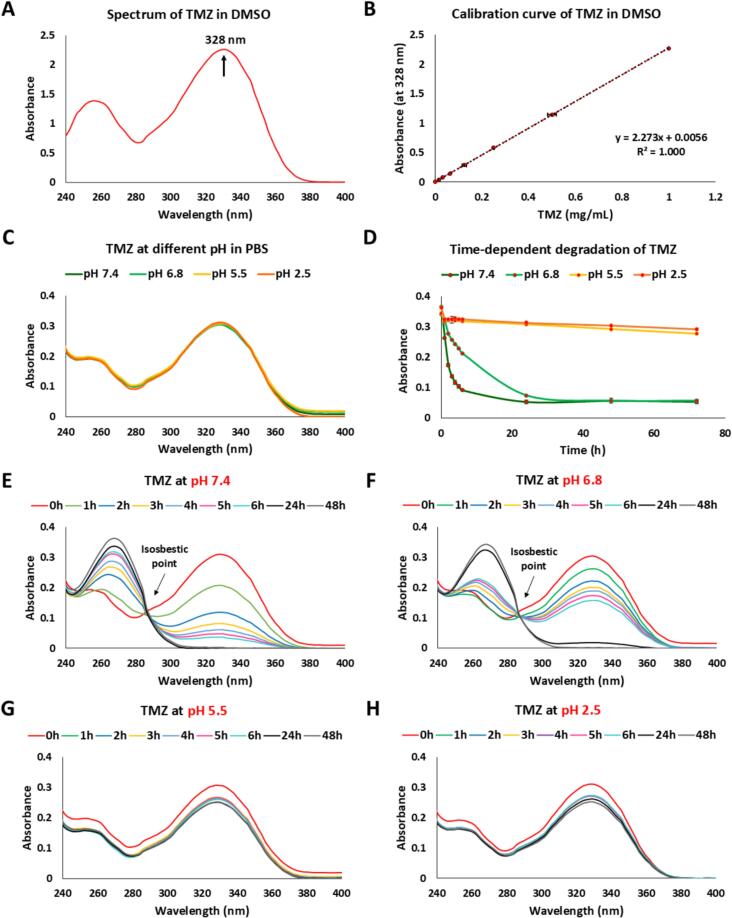
pH-dependent spectral properties and degradation kinetics of TMZ. (A) UV–visible absorption spectrum of TMZ in DMSO with a characteristic peak at 328 nm. (B) Calibration curve of TMZ in DMSO at 328 nm. (C) Absorption spectra of TMZ in PBS at different pH values (7.4, 6.8, 5.5, and 2.5). (D) Time-dependent degradation of TMZ under different pH conditions measured at 328 nm. (E-H) UV–Vis absorption spectra of TMZ in PBS at pH 7.4 (E), pH 6.8 (F), pH 5.5 (G), and pH 2.5 (H) over 48 h. At pH 7.4 and 6.8, a progressive decrease in absorbance at 328 nm and the emergence of an isosbestic point indicate hydrolytic degradation. UV–vis spectra were measured by a BioTek Cytation 3 Cell Imaging Multimode Reader.

Maintaining the stability of TMZ is crucial for ensuring its therapeutic efficacy, as TMZ is highly unstable in aqueous solutions at physiological pH. It undergoes hydrolysis and rapidly converts into its active metabolite, 3-methyl-(triazen-1-yl) imidazole-4-carboxamide (MTIC), which significantly affects its bioavailability. For this reason, we analyzed the stability and degradation kinetics of TMZ under different conditions, with a particular focus on solvent effects, pH-dependent stability, and time-dependent degradation. TMZ was first dissolved in DMSO, as it is a commonly used solvent for hydrophobic drugs and is recommended by manufacturers for initial solubilization. The absorption spectrum of TMZ in DMSO exhibits a prominent peak at 328 nm (Fig. 3A), which is consistent with the π-π* electronic transitions characteristic of its chemical structure. This absorption maximum was used for subsequent quantification of TMZ. A calibration curve was generated by measuring the absorbance of different concentrations of TMZ in DMSO at 328 nm (Fig. 3B). The high correlation coefficients indicate that UV–vis spectrophotometry provides a reliable method for TMZ quantification in solvents. However, TMZ is a prodrug that undergoes spontaneous hydrolysis in aqueous environments, and its degradation rate is highly dependent on pH. Stability of TMZ at different pH values (7.4, 6.8, 5.5, and 2.5) is essential for determining the optimal conditions for its encapsulation and delivery. The absorption spectra of TMZ at different pH values (7.4, 6.8, 5.5, and 2.5) were recorded. As shown in Fig. 3C, the spectral profiles remain largely unchanged across pH conditions at time 0. To further investigate the stability of TMZ under physiologically relevant conditions, time-dependent degradation studies were performed (Fig. 3D). TMZ exhibited rapid degradation at pH 7.4, with a significant reduction in absorbance within the first few hours. A similar but slightly slower degradation pattern was observed at pH 6.8. In contrast, at pH 5.5 and pH 2.5, TMZ remained relatively stable, with only minimal absorbance loss over the experimental period. This finding aligns with the known hydrolysis mechanism of TMZ, wherein it undergoes spontaneous degradation in neutral and basic environments but remains more stable in acidic conditions (Lopes et al., 2013). To further characterize the degradation kinetics, full spectral scans of TMZ were performed at multiple time points under each pH condition (Fig. 3*E*-H). At pH 7.4 (Fig. 3E) and pH 6.8 (Fig. 3F), a progressive decrease in absorbance at 328 nm was observed over time, accompanied by the emergence of an isosbestic point around 280 nm. The presence of an isosbestic point suggests a direct and well-defined conversion of TMZ into its hydrolysis products, likely including MTIC and subsequent metabolites. In contrast, at pH 5.5 (Fig. 3G) and pH 2.5 (Fig. 3H), the absorbance spectra remained largely unchanged over time, confirming the relative stability of TMZ under acidic conditions, which slowed down TMZ hydrolysis and prolonged its stability. These findings are particularly relevant for oral drug delivery, as they suggest that TMZ remains stable in the gastric environment prior to absorption and entry into the systemic circulation. In addition, intranasal delivery strategies offer a promising alternative, providing a noninvasive route that bypasses the BBB, enabling direct drug transport to the central nervous system (CNS) via the olfactory and trigeminal pathways.

### 3.4 Encapsulation of temozolomide in *Citrus limon L.*-derived EVs: a strategy for enhanced drug stability and delivery

**Fig. 4:**
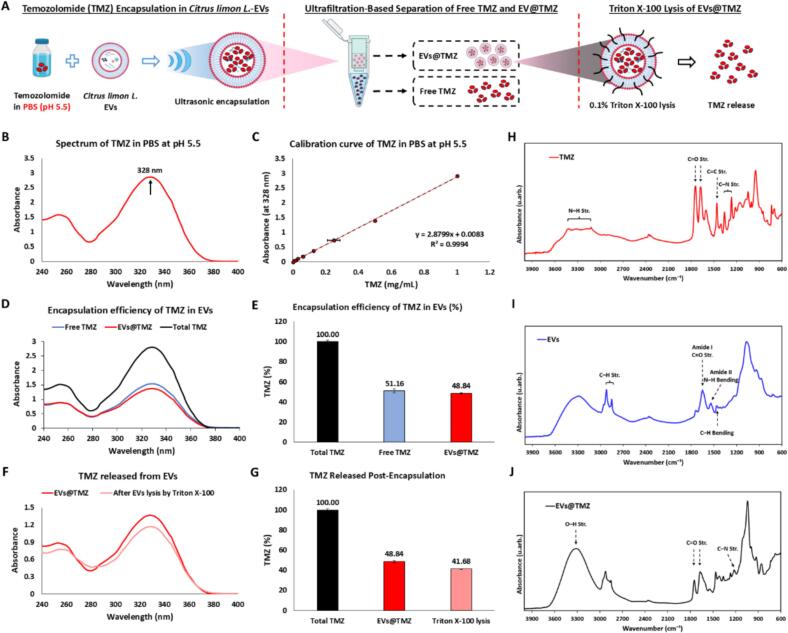
Encapsulation and Release of TMZ in *Citrus limon L.*-Derived EVs. (A) Schematic diagram of TMZ encapsulation in EVs (EVs@TMZ) by ultrasound, separation of free TMZ and EVs@TMZ by ultrafiltration, and release of TMZ after Triton X-100 lysis. (B) UV–Vis absorption spectrum of TMZ in PBS at pH 5.5, with a characteristic peak at 328 nm. (C) Calibration curve of TMZ absorbance at 328 nm in PBS (pH 5.5). (D) UV–Vis absorption spectra of free TMZ, EVs@TMZ, and total TMZ. (E) Encapsulation efficiency of TMZ in EVs, showing the percentage of free TMZ and EVs@TMZ relative to total TMZ. (F) UV–vis spectra of TMZ before and after Triton X-100 lysis of EVs. (G) Quantification of TMZ after EVs lysis. (H–J) Fourier-transform infrared (FTIR) spectra of free TMZ (H), EVs (I), and EVs@TMZ (J) with characteristic functional groups and potential interactions between TMZ and EVs.

Given the short half-life of TMZ and its rapid hydrolysis in physiological fluids, encapsulation strategies such as lipid carriers or EVs-mediated delivery could protect it from premature degradation, thereby enhancing its stability. Natural carriers, compared to synthetic alternatives, offer better biocompatibility, lower immunogenicity, and intrinsic therapeutic properties (e.g., anti-inflammatory and antioxidant effects), which may further support glioblastoma treatment. In this study, we employed *Citrus limon L.*-derived EVs as natural nanocarriers for TMZ encapsulation, extending its half-life and maintaining therapeutic concentrations. To address the cytotoxicity of DMSO at high concentrations, which could compromise EVs integrity, we opted to dissolve TMZ in PBS. Additionally, considering the strong acidity of pH 2.5 limits its biological applicability, we selected pH 5.5, which is more suitable for drug delivery applications. The schematic diagram of TMZ encapsulation in EVs and their release is illustrated in Fig. 4A. The UV–Vis spectrum of TMZ in PBS at pH 5.5 (Fig. 4B) exhibits a characteristic absorption peak at 328 nm, which was used for subsequent quantification. A standard calibration curve was generated by measuring the absorbance of TMZ at 328 nm across different concentrations in PBS at pH 5.5 (Fig. 4C). The encapsulation of TMZ into EVs was carried out through ultrasonication, and the encapsulation efficiency was evaluated by comparing the absorbance spectra of free TMZ (unencapsulated), EVs@TMZ, and total TMZ (Fig. 4D). The analysis revealed that 48.84 % of TMZ was successfully encapsulated within the EVs, while 51.16 % remained unencapsulated in the free form (Fig. 4E). This suggests that EVs can encapsulate a substantial fraction of TMZ, potentially enhancing its delivery properties. To verify the presence of TMZ encapsulated within EVs, the phospholipid membrane of EVs was disrupted using 0.1 % Triton X-100, and the resulting spectral profile was analyzed (Fig. 4F). Our findings revealed that post-lysis led to the release of 41.68 % of TMZ (Fig. 4G), which closely aligned with the encapsulation efficiency shown in Fig. 4E. This finding confirms that the majority of TMZ measured in EVs@TMZ was truly encapsulated rather than adsorbed on the vesicle surface. The consistency between encapsulation and release data highlights the stability of TMZ within EVs. Furthermore, the spectral profiles of free TMZ and EVs@TMZ remained essentially unchanged after one month of the storage at −20 °C, indicating that no degradation occurred (Supplementary Fig. S5). Fourier-transform infrared (FT-IR) spectroscopy revealed the structural characteristics of TMZ, EVs, and EVs@TMZ, providing insights into the interactions between TMZ and EVs (Fig. 4H-J). A broad peak in the 3200–3600 cm^−1^ region, observed in all samples, corresponds to O—H and N—H stretching vibrations. This peak is most pronounced in the EVs@TMZ spectrum, indicating enhanced hydrogen bonding, likely due to the interactions between TMZ and the functional groups present in the EVs. In the region around 1700 cm^−1^, a sharp peak in the TMZ spectrum, characteristic of carbonyl (C=O) stretching (Fig. 4H), shifts and diminishes in intensity in the EVs@TMZ spectrum (Fig. 4J). This change suggests that the carbonyl groups of TMZ interact with components of the EVs, altering their chemical environment. Notably, the amide I (∼1650 cm^−1^) and amide II (∼1540 cm^−1^) peaks, which arise from C=O stretching and N—H bending/C–N stretching in peptide bonds, are prominent in the EVs spectrum (Fig. 4I) but absent in TMZ. The retention and subtle changes of these peaks in EVs@TMZ further indicated the integration of TMZ within the EVs structure. Additionally, lipid-associated peaks provide further evidence of TMZ encapsulation. The C—H stretching vibrations (2800–3000 cm^−1^), representing symmetric and asymmetric stretching of methylene (–CH₂) and methyl (–CH₃) groups in the lipid bilayer, are clearly present in the EVs spectrum. The C—H bending peak (∼1460 cm^−1^), indicative of alkyl chains in lipid molecules, is another distinctive feature of the EVs membrane. These peaks were retained in the EVs@TMZ spectra, reinforcing the preservation of the lipid structure of EVs after TMZ encapsulation. Collectively, these findings demonstrate that *Citrus limon L.*-derived EVs can effectively encapsulate TMZ, serving as natural nanocarriers for chemotherapeutic drug delivery, contributing to improved drug stability, targeted delivery, and therapeutic efficacy.

### 3.5 Dual effects of *Citrus limon. L*-derived EVs: enhancing endothelial cell viability and wound healing, while eliciting cytotoxicity and ROS production in glioblastoma cells

**Fig. 5:**
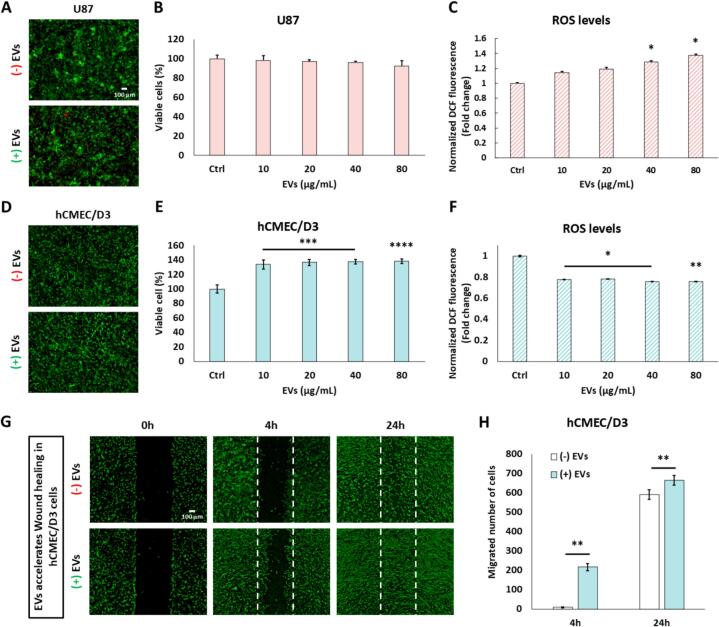
Effects of *Citrus limon L.*-derived EVs on U87 and hCMEC/D3 Cells. (A, D) Representative live/dead staining images of U87 and hCMEC/D3 cells, respectively, following 48-h treatment with EVs (80 μg/mL). Live cells are stained green (Calcein AM), while dead cells are stained red (PI). (B, E) Cell viability of U87 and hCMEC/D3 cells treated with increasing concentrations of EVs (10, 20, 40, and 80 μg/mL). Data are expressed as the percentage of viable cells relative to the control (mean ± SEM, *n* = 4). (C, F) Intracellular ROS levels in U87 and hCMEC/D3 cells were normalized to the number of viable cells. (G) Wound healing assay showing hCMEC/D3 cell migration at 0-, 4-, and 24-h post-scratch, (+) with or (−) without EVs. (H) Quantification of migrating cells and hCMEC/D3 cells after scratch wounding. Statistical significance was determined using one-way or two-way ANOVA with Tukey's post hoc test (*p < 0.05; **p < 0.01; ***p < 0.001; ****p < 0.0001). (For interpretation of the references to colour in this figure legend, the reader is referred to the web version of this article.)

To evaluate the biocompatibility of EVs, we examined their effects on U87 glioblastoma cells and hCMEC/D3 human brain endothelial cells. Live/dead staining and quantitative analysis indicated that EVs exerted a slight cytotoxic effect on U87 cells, though not in a statistically significant manner (Fig. 5A, B), while promoting hCMEC/D3 cell survival in a dose-dependent manner, with significant enhancements observed at 10, 20, 40, and 80 μg/mL (Fig. 5D, E). Increased intracellular ROS levels in U87 cells upon treatment with EVs at 40 and 80 μg/mL (Fig. 5C), indicating increased oxidative stress. In contrast, the ROS levels of hCMEC/D3cells decreased significantly with increasing EVs concentrations, indicating a protective antioxidant effect on brain endothelial cells (Fig. 5F). In view of these interesting results, the potential pro-migratory effects of EVs on hCMEC/D3 cells were evaluated by wound healing assay. Representative images at 0-, 4-, and 24-h post-scratch revealed that EVs accelerated cells migration (Fig. 5G). Quantitative analysis confirmed that the number of migrating cells was significantly increased in the presence of EVs (Fig. 5H). These findings suggest that EVs enhance the migration capacity of brain endothelial cells and may contribute to endothelial repair and vascular remodeling.

### 3.6 EVs@TMZ mitigates endothelial toxicity while preserving anti-tumor and anti-angiogenic effects in a 3D BTB model

**Fig. 6:**
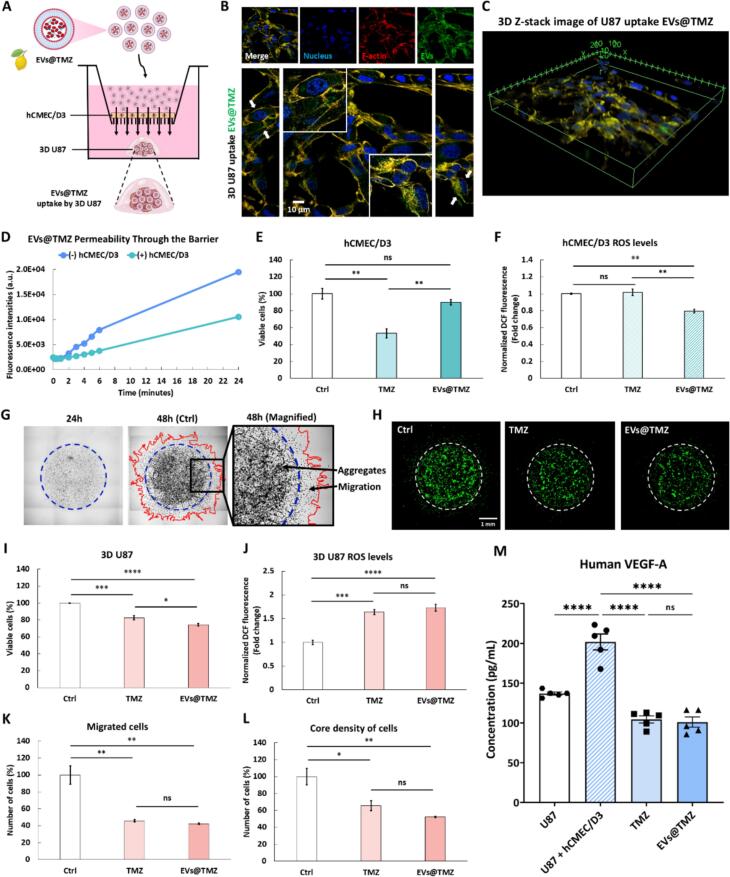
Evaluation of EVs@TMZ uptake, cytotoxicity, glioblastoma spheroid progression, and VEGF-A secretion in a 3D BTB model. (A) Schematic illustration of EVs@TMZ uptake by U87 in a 3D BTB co-culture model with hCMEC/D3 cells. (B) Confocal microscopy images of 3D U87 cells incubated with EVs@TMZ at 37 °C for 24 h. EVs@TMZ were labeled with calcein-AM (shown in green), actin filaments were labeled with rhodamine phalloidin, and nuclei were counterstained with 4′,6-diamidino-2-phenylindole (DAPI) (scale bar = 10 μm). The magnified images depict high-magnification views of the white boxed areas. (C) 3D *Z*-stack image of spatial distribution and uptake of EVs@TMZ in U87 cells. (D) Progressive BTB crossing of EVs@TMZ over 24 h. (E) Cytotoxic effects and (F) ROS levels in hCMEC/D3 cells after 48 h of untreated or treatment with TMZ and EVs@TMZ. (G) Representative stitched microscopic images of 3D U87 in the untreated (Ctrl) group at 24 and 48 h, with arrows indicating aggregates and migration. (H) Representative fluorescence images of 3D U87 after 48 h under three conditions: untreated (Ctrl), TMZ-treated, and EVs@TMZ-treated. (I-L) Quantification of 3D U87, including viable cells (I), ROS levels (J), migrated cells (K), and core density (L). (M) VEGF-A levels in U87, U87 co-cultured with hCMEC/D3 (U87 + hCMEC/D3), TMZ-treated, and EVs@TMZ-treated. Statistical significance was determined using one-way ANOVA with Tukey's post hoc test (*p < 0.05; **p < 0.01; ***p < 0.001; ****p < 0.0001). (For interpretation of the references to colour in this figure legend, the reader is referred to the web version of this article.)

To assess whether EVs encapsulation reduces the off-target toxicity of TMZ while maintaining its therapeutic efficacy, a 3D BTB model was established. First, hCMEC/D3 endothelial cells were pre-cultured on the bottom side of a transwell insert for 7 days to develop tight junctions, forming a physiologically relevant barrier. Next, U87 glioblastoma cells were bioprinted to mimic the tumor microenvironment (Fig. 6A). This transwell-based 3D co-culture system was then applied to evaluate the permeability of EVs@TMZ across the BTB. The uptake of EVs@TMZ by 3D U87 cells was confirmed by confocal imaging (Fig. 6B), where fluorescently labeled EVs (green) were observed within the cytoplasm, as further demonstrated by 3D *Z*-stack reconstruction, highlighting their efficient internalization (Fig. 6C). To assess EVs@TMZ permeability across the endothelial barrier, the EVs were stained with Calcein-AM, and their fluorescence signal was monitored over 24 h (Fig. 6D). The results demonstrated a progressive increase in fluorescence intensity, indicating effective permeation across the endothelial barrier and suggesting enhanced drug accumulation within the tumor microenvironment. To further evaluate the impact of this delivery system, the cytotoxic effects of TMZ and EVs@TMZ were assessed in hCMEC/D3 endothelial cells. TMZ treatment led to a notable reduction in cell viability compared to untreated control, indicating substantial endothelial toxicity. In contrast, EVs@TMZ treatment preserved cell viability to a greater extent than TMZ alone, with only a slight decrease relative to the control, suggesting that EVs encapsulation mitigates TMZ-induced endothelial damage (Fig. 6E). To further examine endothelial damage, oxidative stress levels were measured under different treatment conditions. While no statistically significant difference was observed, the TMZ-treated group exhibited a slight increase in ROS levels compared to the control. Meanwhile, EVs@TMZ treatment effectively reduced ROS accumulation, demonstrating that EVs encapsulation alleviates TMZ-induced oxidative damage while potentially preserving endothelial cell homeostasis (Fig. 6F). Beyond its attenuated effect on endothelial cells, EVs@TMZ were also evaluated for their impact on GBM cell growth and invasion. Brightfield images at 24- and 48-h post-treatment (Fig. 6G) revealed that untreated control group exhibited pronounced cell aggregation and migration (marked by red contours), whereas in both the TMZ and EVs@TMZ treatment groups, cell migration was obviously suppressed, leading to a denser, more compact structure (Fig. 6H). Quantitative analysis of glioblastoma proliferation (Fig. 6I) demonstrated a significant reduction in the total number of U87 cells following TMZ and EVs@TMZ treatment compared to the control group. Consistently, both treatments induced oxidative stress, as evidenced by increased ROS levels (Fig. 6J). Likewise, glioblastoma cell migration (Fig. 6K) and core cell density in the model (Fig. 6L) were significantly lower in both treatment groups, with EVs@TMZ showing comparable efficacy to TMZ in inhibiting glioblastoma invasion. To further explore the impact of treatment on glioblastoma-endothelial interactions, VEGF-A secretion, a key pro-angiogenic factor, was quantified. Co-culturing U87 glioblastoma cells with hCMEC/D3 endothelial cells led to a substantial increase in VEGF-A secretion compared to U87 monoculture, highlighting the strong pro-angiogenic nature of the GBM microenvironment. TMZ treatment markedly reduced VEGF-A levels, indicating its effectiveness in suppressing glioblastoma-driven angiogenesis. Similarly, EVs@TMZ treatment demonstrated a comparable inhibitory effect, confirming that the encapsulation of TMZ in EVs does not compromise its anti-angiogenic properties (Fig. 6M).

## 4 Discussion

The treatment of GBM is hindered by the challenges of drug stability, poor BBB penetration, and severe systemic toxicity. Recent advancements in EVs-based drug delivery have highlighted their potential as natural, biocompatible carriers for targeted therapies. Though mammalian EVs have been extensively explored, concerns about their immunogenicity, heterogeneity, and production costs have led to increasing interest in PDEVs, which offer advantages such as low immunogenicity, large-scale production, and inter-kingdom communication capabilities.

Drug loading remains a critical factor in optimizing EVs-based delivery. Conventional loading techniques, such as sonication, electroporation, and incubation, have been explored to enhance drug retention within EVs (Du et al., 2023; Jiang et al., 2024; Yi et al., 2025). However, the instability of TMZ, characterized by its short half-life due to rapid hydrolysis in physiological fluids, poses significant challenges for formulation and therapeutic application (Amarandi et al., 2022). To enhance the stability of TMZ, we optimized its dissolution pH to 5.5, which effectively slowed down the hydrolysis and prolonged its stability. Encapsulation approaches, such as EVs-mediated delivery, play a crucial role in protecting TMZ, extending its circulation time, and ensure sustained drug release at the tumor site, ultimately improving therapeutic efficacy. Liposomal-based formulations have been widely explored for TMZ delivery, yet their clinical translation remains limited due to issues such as rapid clearance, systemic toxicity, and suboptimal BBB penetration (Juhairiyah and de Lange, 2021). PDEVs, on the other hand, represent a natural and versatile alternative with intrinsic bioactivity, potentially offering a more effective means of delivering TMZ to GBM tumors. Their natural composition allows for efficient encapsulation and protection of both hydrophilic and hydrophobic drugs, ensuring stability and bioactivity during systemic circulation. Our study achieved high encapsulation efficiency of TMZ in PDEVs, aligning with previous reports on exosome-based drug delivery systems, including the efficient loading of doxorubicin into ginger-derived EVs (Zhang et al., 2016).

Crossing the BBB remains a major hurdle in GBM treatment, limiting the efficacy of chemotherapeutic agents. Traditional chemotherapeutic approaches often compromise BBB integrity, leading to adverse effects. A key advantage of EVs is their ability to traverse the BBB, primarily through mechanisms such as receptor-mediated endocytosis and micropinocytosis. This capability is attributed to their endogenous lipid composition and inherent cellular uptake pathways, facilitating effective intracellular drug delivery (Ramos-Zaldívar et al., 2022). To assess this capability, we employed an advanced 3D BTB model to evaluate the permeability of EVs@TMZ, capable of faithfully resembling in vivo tumor surrounding environment. PDEVs demonstrated efficient BBB penetration in the 3D model while protecting endothelial cells by preserving vascular integrity and reducing ROS accumulation. This selective ROS modulation may be attributed to differences in cellular uptake, the presence of intrinsic antioxidant compounds within the PDEVs, or cell-specific TMZ release kinetics. Although these mechanisms were not directly explored in this study, they warrant further investigation. Future work should examine these possibilities through quantitative uptake studies, biochemical profiling of vesicle contents, and time-resolved analysis of drug release in different cell types to clarify the molecular basis of this context-dependent effect. By limiting exposure to healthy tissues, PDEVs-mediated drug encapsulation has the potential to reduce side effects, lower the required therapeutic dosage, and mitigate systemic toxicity. Given the critical role of BBB integrity in CNS homeostasis and prevention of neuroinflammation, the high biocompatibility of EVs with endothelial cells highlights their promise as safe and effective delivery vehicles for CNS-targeted therapeutics. Although this study focused on TMZ monotherapy, the PDEV platform offers substantial flexibility for combinatorial strategies aimed at improving therapeutic efficacy. Co-delivery of complementary agents—such as anti-angiogenic drugs (e.g., bevacizumab), immune checkpoint inhibitors (e.g., anti-PD-1/PD-L1 antibodies), or gene-silencing molecules (e.g., siRNAs targeting MGMT or VEGF)—could synergistically target multiple components of the tumor microenvironment. Given their favorable delivery characteristics and natural origin, PDEVs represent a promising nanocarrier system for the development of multifunctional therapeutic formulations. Future studies will investigate such combinations to broaden the translational potential of this approach.

Beyond serving as drug carriers, PDEVs possess inherent bioactive properties that may contribute to GBM therapy. Our findings demonstrate that EVs@TMZ maintain cytotoxic effects against U87 glioblastoma cells while preserving endothelial cell integrity, highlighting their dual therapeutic potential. The selective toxicity toward glioblastoma cells is likely attributed to the ability of EVs to encapsulate and efficiently deliver TMZ, enhancing drug efficacy while minimizing off-target effects. Additionally, the observed VEGF-A downregulation following EVs@TMZ treatment suggests disruption of key pro-tumorigenic pathways, leading to impaired angiogenesis. However, whether this effect is driven solely by TMZ-induced cytotoxicity or partially mediated by the intrinsic bioactive cargo of PDEVs remains to be elucidated. Although this study focused on functional outcomes in a 3D BTB model, future transcriptomic or proteomic analyses of PDEVs and treated cells would be instrumental in uncovering the molecular mechanisms involved in VEGF-A regulation and the broader anti-angiogenic response. By sustaining cytotoxic effects and modulating the tumor microenvironment, EVs-mediated TMZ delivery inhibits tumor growth while simultaneously disrupting its supportive vasculature, thereby reinforcing its therapeutic advantage in glioblastoma treatment. Despite these promising findings, several challenges must be addressed before clinical translation. In particular, the precise mechanisms underlying PDEVs-mediated transport across the blood–brain and blood–tumor barriers require further elucidation through molecular and in vivo imaging studies. Moreover, although our advanced 3D BTB model provides a physiologically relevant platform to assess permeability and efficacy, the lack of in vivo validation represents a current limitation. Future studies will involve pharmacokinetic, biodistribution, and therapeutic evaluations in orthotopic glioblastoma mouse models to confirm tumor accumulation, systemic safety, and treatment efficacy under physiologically complex conditions. These investigations will be essential to substantiate the translational potential of this plant-derived delivery platform.

## 5 Conclusion

This study highlights the potential of *Citrus limon L.*-derived EVs as a natural and efficient nanocarrier for TMZ delivery in glioblastoma treatment. Using a physiologically relevant 3D BTB model, we demonstrate that PDEVs successfully cross the BBB while preserving endothelial integrity, mitigating oxidative stress, and reducing TMZ-induced toxicity. EVs@TMZ enhances drug stability, promotes targeted tumor delivery, and maintains potent cytotoxicity against GBM cells while minimizing off-target effects on healthy brain vasculature. Furthermore, its ability to modulate the tumor microenvironment by inhibiting angiogenesis through downregulation of VEGF-A highlights its dual therapeutic potential in tumor suppression and vascular homeostasis. By overcoming key limitations such as poor BBB permeability and systemic toxicity, PDEVs present a promising, biocompatible platform for brain tumor therapy. These findings support further investigation of PDEV as a naturally derived drug delivery system with the potential to improve therapeutic efficacy and clinical outcomes in GBM patients.

## CRediT authorship contribution statement

**Lishan Cui:** Writing – review & editing, Writing – original draft, Methodology, Investigation, Formal analysis, Data curation. **Giordano Perini:** Writing – original draft, Visualization, Validation, Formal analysis, Data curation. **Antonio Minopoli:** Writing – original draft, Validation, Software, Formal analysis. **Valentina Palmieri:** Writing – original draft, Supervision, Investigation, Formal analysis. **Marco De Spirito:** Writing – original draft, Visualization, Validation, Formal analysis. **Massimiliano Papi:** Writing – review & editing, Writing – original draft, Supervision, Resources, Project administration, Funding acquisition, Formal analysis.

## Ethics statement

Ethical approval was not required for this study in accordance with the local legislation and institutional requirements because only commercially available established cell lines were used.

## Funding

The author(s) declare that financial support was received for the research, authorship, and/or publication of this article. This work was supported by the 10.13039/501100004710Fondazione Umberto Veronesi (Postdoctoral Fellowships Grant 2024 to LC).

## Declaration of competing interest

The authors declare that the research was conducted in the absence of any commercial or financial relationships that could be construed as a potential conflict of interest.

## Data Availability

The original contributions presented in the study are included in the article/Supplementary Material, further inquiries can be directed to the corresponding authors.

## References

[bb0005] Alzahrani F.A., Khan M.I., Kameli N., Alsahafi E., Riza Y.M. (2023). Plant-derived extracellular vesicles and their exciting potential as the future of next-generation drug delivery. Biomolecules.

[bb0010] Amarandi R.-M., Ibanescu A., Carasevici E., Marin L., Dragoi B. (2022). Liposomal-based formulations: a path from basic research to temozolomide delivery inside glioblastoma tissue. Pharmaceutics.

[bb0015] Auffinger B., Tobias A.L., Han Y., Lee G., Guo D., Dey M., Lesniak M.S., Ahmed A.U. (2014). Conversion of differentiated cancer cells into cancer stem-like cells in a glioblastoma model after primary chemotherapy. Cell Death & Differentiation.

[bb0020] Berumen Sánchez G., Bunn K.E., Pua H.H., Rafat M. (2021). Extracellular vesicles: mediators of intercellular communication in tissue injury and disease. Cell Communication and Signaling.

[bb0025] Cai Q., Li X., Xiong H., Fan H., Gao X., Vemireddy V., Margolis R., Li J., Ge X., Giannotta M., Hoyt K., Maher E., Bachoo R., Qin Z. (2023). Optical blood-brain-tumor barrier modulation expands therapeutic options for glioblastoma treatment. Nat. Commun..

[bb0030] Chamberlain M.C. (2010). Temozolomide: therapeutic limitations in the treatment of adult high-grade gliomas. Expert Rev. Neurother..

[bb0035] Cui L., Perini G., Augello A., Palmieri V., De Spirito M., Papi M. (2024). Plant-derived extracellular nanovesicles: a promising biomedical approach for effective targeting of triple negative breast cancer cells. Front. Bioeng. Biotechnol..

[bb0040] Cui L., Perini G., Minopoli A., Augello A., De Spirito M., Palmieri V., Papi M. (2024). Plant-derived extracellular vesicles release combined with systemic DOX exhibits synergistic effects in 3D bioprinted triple-negative breast cancer. Biomed. Pharmacother..

[bb0045] Du S., Guan Y., Xie A., Yan Z., Gao S., Li W., Rao L., Chen X., Chen T. (2023). Extracellular vesicles: a rising star for therapeutics and drug delivery. J. Nanobiotechnol..

[bb0050] Elsharkasy O.M., Nordin J.Z., Hagey D.W., de Jong O.G., Schiffelers R.M., Andaloussi S.E.L., Vader P. (2020). Extracellular vesicles as drug delivery systems: why and how?. Adv. Drug Deliv. Rev..

[bb0055] Gomez-Roman N., Stevenson K., Gilmour L., Hamilton G., Chalmers A.J. (2017). A novel 3D human glioblastoma cell culture system for modeling drug and radiation responses. Neuro Oncol..

[bb0060] Jiang S., Cai G., Yang Z., Shi H., Zeng H., Ye Q., Hu Z., Wang Z. (2024). Biomimetic nanovesicles as a dual gene delivery system for the synergistic gene therapy of Alzheimer’s disease. ACS Nano.

[bb0065] Juhairiyah F., de Lange E.C.M. (2021). Understanding drug delivery to the brain using liposome-based strategies: studies that provide mechanistic insights are essential. AAPS J..

[bb0070] Kürtösi B., Kazsoki A., Zelkó R. (2024). A systematic review on plant-derived extracellular vesicles as drug delivery systems. Int. J. Mol. Sci..

[bb0075] Lian M.Q., Chng W.H., Liang J., Yeo H.Q., Lee C.K., Belaid M., Tollemeto M., Wacker M.G., Czarny B., Pastorin G. (2022). Plant-derived extracellular vesicles: recent advancements and current challenges on their use for biomedical applications. J Extracell Vesicles.

[bb0080] Lopes I.C., de Oliveira S.C.B., Oliveira-Brett A.M. (2013). Temozolomide chemical degradation to 5-aminoimidazole-4-carboxamide – electrochemical study. J. Electroanal. Chem..

[bb0085] Marar C., Starich B., Wirtz D. (2021). Extracellular vesicles in immunomodulation and tumor progression. Nat. Immunol..

[bb0090] Miller D.S., Bauer B., Hartz A.M.S. (2008). Modulation of P-glycoprotein at the blood-brain barrier: opportunities to improve central nervous system pharmacotherapy. Pharmacol. Rev..

[bb0095] Nagelkerke A., Ojansivu M., van der Koog L., Whittaker T.E., Cunnane E.M., Silva A.M., Dekker N., Stevens M.M. (2021). Extracellular vesicles for tissue repair and regeneration: evidence, challenges and opportunities. Adv. Drug Deliv. Rev..

[bb0100] Ramos-Zaldívar H.M., Polakovicova I., Salas-Huenuleo E., Corvalán A.H., Kogan M.J., Yefi C.P., Andia M.E. (2022). Extracellular vesicles through the blood-brain barrier: a review. Fluids Barriers CNS.

[bb0105] Saeedi S., Israel S., Nagy C., Turecki G. (2019). The emerging role of exosomes in mental disorders. Transl. Psychiatry.

[bb0110] Weksler B., Romero I.A., Couraud P.-O. (2013). The hCMEC/D3 cell line as a model of the human blood brain barrier. Fluids and Barriers of the CNS.

[bb0115] Witwer K.W., Wolfram J. (2021). Extracellular vesicles versus synthetic nanoparticles for drug delivery. Nat. Rev. Mater..

[bb0120] Wu D., Chen Q., Chen X., Han F., Chen Z., Wang Y. (2023). The blood–brain barrier: structure, regulation and drug delivery. Signal Transduct. Target. Ther..

[bb0125] Xu Z., Xu Y., Zhang K., Liu Y., Liang Q., Thakur A., Liu W., Yan Y. (2023). Plant-derived extracellular vesicles (PDEVs) in nanomedicine for human disease and therapeutic modalities. J. Nanobiotechnol..

[bb0130] Yi W., Hu S., Qian X., Yan W., Li Y. (2025). Synthetic biology-based engineering cells for drug delivery. Exploration (Beijing).

[bb0135] Zhang M., Xiao B., Wang H., Han M.K., Zhang Z., Viennois E., Xu C., Merlin D. (2016). Edible ginger-derived nano-lipids loaded with doxorubicin as a novel drug-delivery approach for colon cancer therapy. Mol. Ther..

[bb0140] Zhou X., Xie F., Wang L., Zhang L., Zhang S., Fang M., Zhou F. (2020). The function and clinical application of extracellular vesicles in innate immune regulation. Cell. Mol. Immunol..

